# Can postponement of an adverse outcome be used to present risk reductions to a lay audience? A population survey

**DOI:** 10.1186/1472-6947-7-8

**Published:** 2007-03-29

**Authors:** Rasmus Dahl, Dorte Gyrd-Hansen, Ivar Sønbø Kristiansen, Jørgen Nexøe, Jesper Bo Nielsen

**Affiliations:** 1 Research Unit of General Practice, University of Southern Denmark Odense, Denmark; 2 Institute of Public Health, University of Southern Denmark, Odense, Denmark; 3 Institute of Health Management and Health Economics, University of Oslo, Norway

## Abstract

**Background:**

For shared decision making doctors need to communicate the effectiveness of therapies such that patients can understand it and discriminate between small and large effects. Previous research indicates that patients have difficulties in understanding risk measures. This study aimed to test the hypothesis that lay people may be able to discriminate between therapies when their effectiveness is expressed in terms of postponement of an adverse disease event.

**Methods:**

In 2004 a random sample of 1,367 non-institutionalized Danes aged 40+ was interviewed in person. The participants were asked for demographic information and asked to consider a hypothetical preventive drug treatment. The respondents were randomized to the magnitude of treatment effectiveness (heart attack postponement of 1 month, 6 months, 12 months, 2 years, 4 years and 8 years) and subsequently asked whether they would take such a therapy. They were also asked whether they had hypercholesterolemia or had experienced a heart attack.

**Results:**

In total 58% of the respondents consented to the hypothetical treatment. The proportions accepting treatment were 39%, 52%, 56%, 64%, 67% and 73% when postponement was 1 month, 6 months, 12 months, 2 years, 4 years and 8 years respectively. Participants who thought that the effectiveness information was difficult to understand, were less likely to consent to therapy (*p *= 0.004).

**Conclusion:**

Lay people can discriminate between levels of treatment effectiveness when they are presented in terms of postponement of an adverse event. The results indicate that such postponement is a comprehensible measure of effectiveness.

## Background

Prevention and treatment of chronic disease processes such as atherosclerosis, diabetes and osteoporosis represent an important challenge for health care systems in most countries. Preventive interventions, however, often need to be long lasting or even life long in order to realize their full potential [1]. There is evidence that many patients discontinue therapy because they are not convinced about their need for treatment, consider the efficacy to be poor or experience adverse events [2]. Also, patients with dyslipidaemia are more likely to discontinue medication in primary than in secondary prevention [3]. This pattern of discontinuation may partly reflect lack of ability to judge the effectiveness amongst patients. It is therefore important to develop quantitative measures for risk reduction such that clinicians as well as lay people understand them.

There is evidence that involvement of the patient in decisions about therapy increases adherence to therapies for chronic diseases [4,5]. Shared decision making, however, requires that patients are able to understand and respond to the information provided by the doctor. This is not an easy task in the context of chronic disease processes because patients cannot judge the effectiveness from their own experiences. Traditionally, effectiveness has been expressed in risk terms such as absolute risk reduction (ARR), its reciprocal the number needed to treat (NNT) or relative risk reduction (RRR). There is an increasing body of evidence to suggest that preferences for or opinions about a therapy depends on the way it is presented. A therapy may be judged as more favorable when it is presented in terms of RRR than ARR or NNT even when the underlying effectiveness is the same [6-8]. This could indicate that these risk measures are difficult to comprehend. Indeed, there is evidence that lay people and professionals may have difficulties understanding risk measures [9-12]. More recently, natural frequencies and pictorial representation have shown promising results in terms of comprehensibility [9].

When an intervention (*e.g*. life style change or pharmaceutical) reduces the risk of adverse outcomes, it implies that these adverse outcomes are postponed or even avoided. While fatal outcomes cannot be avoided in the long run, outcomes such as myocardial infarction may be postponed to the extent that the patient dies from another cause before he/she experiences them. Postponements of adverse outcomes (death, heart attack, hip fracture, *etc*.) are therefore an alternative way of expressing the effectiveness of interventions for chronic disease processes. Such postponements can be estimated on the basis of clinical trials where the area between the (event free) survival curves represents the mean postponement. It is conceivable that people understand postponement of adverse outcomes better than risk reductions because we are used to judging time and can easily distinguish for example one week from one month or one year. The benefit in terms of average postponement of hip fractures from osteoporosis intervention was estimated at 12 days when an intervention that reduces the risk of hip fracture by 50%, was initiated at the age of 50 and continued for one year [13]. If treatment was initiated at ages 60, 70, 80 or 90 years average postponement of hip fracture would be 23, 55, 90, and 74 days respectively. If treatment lasted for 10 years the average postponement was estimated at 146, 260, 369, 373, and 167 days respectively [13]. In a later study the findings suggested that laypersons were able to discriminate between levels of osteoporosis intervention effectiveness when it was presented in terms of hip fracture postponement rather than NNT [14].

In the Scandinavian Simvastatin Survival Study [15] NNT to avoid one case of myocardial infarction or heart attack was nine over the 5.4 years median follow-up period. This may be translated into an average postponement of myocardial infarction of 3 months [16].

The aim of the present study was to explore whether lay people are able to understand information on intervention effectiveness in the sense that they are able to discriminate between different magnitudes of postponement. The hypothesis was that lay people's preferences for a preventive therapy against heart attack were influenced by the magnitude of its effect when they are expressed in terms of postponement of disease.

## Methods

A random sample of non-institutionalised individuals aged 40 or more was invited to an in-person interview in the spring of 2004. The sample was drawn from Statistics Denmark's list of inhabitants. The population was divided in 42 geographic strata, and respondents were drawn from each stratum to ensure geographic representativity. The interviews were performed by Gallup TNS as part of its interview business, and no specific ethical approval was required. Interviews were undertaken in the respondents' homes, and up to three calls were made to obtain an interview. The total sample was 3,111 persons. Of these individuals 54 (2%) were not fluent in Danish, 136 (4%) were ill, demented or had hearing impairment and 178 (6%) had addresses that were non-inhabited, non-existing or used for industrial purposes. Of the remaining 2,743 individuals, 591 (22%) could not be contacted and 785 (28%) refused to participate. In total 1,367 of 2,743 (50%) subjects were interviewed in their homes.

The respondents were asked for sociodemographic background variables such as gender, age, education and household income. In addition they were asked whether they had experienced a heart attack or were diagnosed with hypercholesterolemia. Eventually the interviewer presented one of the two following statements orally and written on a card:

**A (the extended version)**: "Imagine that your physician tells you that you have a somewhat increased cholesterol level, and that on average 50% of all patients with a similar cholesterol level will suffer a heart attack during their lifespan. You can not predict which patient will suffer a heart attack. Heart attack is rarely seen in patients less than 50 years old and the majority of patients are more than 70 years old.

The physician presents you with a medication which should be taken once a day. The medication has only few and harmless side effects. If you take the treatment, you will need to visit your doctor once a year. The medication will cost you DKK 500 (€1.00≈7, 54 DKK) per year.

The medication postpones a heart attack by approximately (for example) 1 month, if you continue the treatment for the rest of your life.

Would you choose to accept such treatment?"

Or

**B (the limited version)**: "Imagine that your physician tells you that you have a somewhat increased cholesterol level and an increased risk of suffering a heart attack. The physician presents you with a medication which should be taken once a day. The medication has only few and harmless side effects. If you take the treatment, you will need to visit your doctor once a year. The medication will cost you DKK 500 per year.

The medication postpones a heart attack by approximately (for example) 1 month if you continue the treatment for the rest of your life.

Would you choose to accept such treatment?"

The respondents could answer "yes", "no" or "not certain/do not know".

The respondents were randomly assigned to postponement of either 1 month, 6 months, 12 months, 2 years, 4 years or 8 years. The randomization was performed at the interview by means of a computerized system, and the study was designed with the sole purpose of exploring whether lay people can discriminate between levels of effectiveness when they are presented in terms of postponement of adverse outcomes.

Similar wording has been used in several other studies, and has also been tested in focus groups [10,14,17]. An extended and a limited introductory text was presented in order to test whether more detailed information on the actual distribution of benefits and the uncertainty regarding the effect of medication for the individual patient would change preference patterns.

Subsequently, the respondents were asked whether it was difficult to understand the effectiveness of the medication (not difficult, a little difficult, very difficult or impossible to understand).

The data were analyzed using the STATA statistical package version 8.0. We tested the hypothesis that increasing the magnitude of postponement of the heart attack would increase the respondents' consent to therapy. Consent was present if the subject answered yes to treatment and not present if the subject rejected the treatment or stated too little information was given. We used logistic regression analysis to explore the association between consent to therapy and the independent variables. We used the log with base 2 of number of years postponement because a doubling in postponement equals + 1 on this scale.

## Results

The mean age of the 1,367 respondents was 60 years, 52% were female, 74% had education beyond elementary school, and the median family income was in the range DKK200,000 to DKK400,000 (Table 1). The age and gender distribution was similar to that of the Danish population while the respondents had a lower proportion of individuals with no more than elementary schooling (26% *versus *32% in the general population).

On average 58% of all respondents accepted the hypothetical drug treatment, 30% rejected it and 12% were undecided (Table 2). Amongst those respondents who received extended information the proportion who accepted was 54% while 62% accepted therapy when presented with the limited information (*p *= 0.004). Amongst all respondents the proportions accepting the treatment were 39%, 52%, 56%, 64%, 67% and 73% for postponement of a heart attack of 1 month, 6 months, 12 months, 2 years, 4 years and 8 years, respectively (Table 2). These results indicate a trend towards greater acceptance for larger postponements (for statistical test see table 3), but the incremental increase in the proportions accepting diminished with increasing postponement (Table 2).

Previous heart attack was reported by 5% of the respondents, and 18% reported hypercholesterolemia. These respondents were more likely to accept the hypothetical treatment than their counterparts without personal experience with these diseases (*p *= 0.008 and *p *< 0.001).

In total 81% of the respondents indicated that the effectiveness information was not difficult to understand, while others found it somewhat difficult (14%), very difficult (4%) or impossible to understand (2%) (Table 2). There was no difference in level of understanding across postponement groups. Respondents who had no difficulties in understanding the information were more likely to accept the hypothetical treatment (*p *≤ 0.001) (table 3). This trend was seen in the group given the limited version of information as well as in the group given the extended information. Presentation of extended information did not impact on perceived understanding of the information (chi^2 ^= 0.102; *p *= 0.992).

The results were confirmed in a logistic regression analysis of consent to therapy (Table 3). Here, male gender (odds ratio (OR) 0.77, 95% confidence interval (CI) 0.61–0.97), level of understanding, elevated cholesterol level (OR 1.85, CI 1.75–2.58) and previous myocardial infarction (OR 2.53, CI 1.28–5.00) increased the odds for consenting to therapy. The odds also increased with increasing effectiveness of the therapy (postponement of heart attack), but decreased when extended information were presented. The effect of age on consent to therapy was inconsistent, and there was no significant effect of educational attainment. Household income was omitted from the regression analysis due to multi-collinearity.

## Discussion

In this randomized study, the participants could discriminate between different levels of effectiveness when they were presented in terms of postponement of a heart attack. The results may indicate that postponement of adverse health outcomes can be used to convey information about treatment effectiveness for lay people.

This interpretation, however, should be seen against the limitations of the study. First the response rate was relatively low (50%), and data on background variables indicate that the study sample was not entirely representative of the Danish population. The internal validity, however, may be good in that the groups were balanced in terms of background variables. Secondly we can only observe that the participants could discriminate between different levels of effectiveness, but we do not know how they interpreted this effectiveness information. The participants may assume that the postponement is "tacked" onto a disease-free average lifespan [18] not realizing that the gain of the preventive intervention could be in the near future. Likewise, respondents may perceive the postponement as "guaranteed" not realizing that one cannot predict the benefit to the individual patient. The extended information addresses these issues, and the reduction in propensity to participate observed amongst those respondents presented with this added information, suggests that some individuals may be steered by such miscomprehensions.

The findings in our study accord with another study [14] demonstrating that lay people seem to discriminate between treatment effectiveness when it is expressed as postponement of hip fracture. It is also seen that participants with hypercholesterolemia or previous heart attack are more likely to accept the treatment supporting the findings of previous studies that show that patients in secondary prevention are more likely to continue therapy [3]. Our results, however, are in contrast to previous studies which show that lay people have difficulties in discriminating between levels of effectiveness when they are presented in terms of NNT [10,11,17]. The explanation of this discrepancy may be that risk reductions are generally difficult to comprehend, and respondents may be misled by the so-called "evaluability heuristics". This implies that they base their decisions on cues that they are able to understand (*e.g*. cost of therapy or side effects) rather than information that is difficult to evaluate (*e.g*. treatment effectiveness presented as risk reductions). In contrast, most people can relate to time and differences in time.

Whether postponement of adverse health outcomes can be a useful tool for shared decision making remains to be shown in studies undertaken in a clinical context. The benefits of interventions are unlikely to be evenly spread among patients. The distribution of health gains is generally more complex and cannot be adequately described by way of postponement or risk reductions. Ideally, we need a two dimensional measure which can describe the probability of gain as well as the postponement of adverse events amongst those who gain. However, survival curves from clinical trials do not provide us with such detailed knowledge of the distribution of health benefits. Simulation tests (not shown here) indicate that survival curves have similar appearance irrespective of whether all patients gain 6 months or 1 in 10 patients gain 60 months. One might suspect that patients' preferences are not equal for these two alternatives. Also, it should be noted that some patients may be discouraged when they learn that most health interventions prolong life by less than 12 months [19]. Finally, people may find it odd to think about the therapeutic benefits in terms of postponement of heart attack or hip fracture. The widespread use of the term prevention would indicate that the adverse outcomes are avoided rather than postponed.

## Conclusion

We conclude that postponement of adverse health outcomes is an alternative measure of effectiveness that seems to allow laypersons to discriminate between levels of effectiveness. More research is needed to explore whether postponement of adverse events in practice is useful for shared decision making.

## Abbreviations

ARR: Absolute risk reduction.

NNT: Number needed to treat.

RRR: Relative risk reduction.

## Competing interests

The author(s) declare that they have no competing interests.

## Authors' contributions

RD analysed the data and drafted the manuscript. DGH, ISK, JN and JBN designed the study. All authors were involved in interpreting the findings, revising the manuscript and approve the final version.

## Pre-publication history

The pre-publication history for this paper can be accessed here:



## Figures and Tables

**Table 1:** Characteristics of respondents randomly allocated to different magnitudes of treatment effectiveness expressed in terms of postponement of heart attack. Percentages in parentheses

		**Postponement of heart attack**							
		1 month	6 months	12 months	2 years	4 years	8 years	Total	Danish population*
	Total	226	220	243	244	214	220	1367	2669018
**Age**	40–49	53 (23)	60 (27)	69 (28)	80 (33)	58 (27)	52 (24)	372 (27)	776802 (29)
	50–59	61 (27)	69 (31)	60 (25)	63 (26)	44 (21)	59 (27)	356 (26)	743971 (28)
	60–69	57 (25)	44 (20)	51 (21)	37 (15)	56 (26)	47 (21)	292 (21)	574939 (22)
	70–79	37 (16)	28 (13)	42 (17)	46 (19)	32 (15)	37 (17)	222 (16)	351450 (13)
	80+	18 (8)	19 (9)	21 (9)	18 (7)	24 (11)	25 (11)	125 (9)	221856 (8)

**Gender**	Women	108 (48)	121 (55)	130 (53)	124 (51)	109 (51)	125 (57)	717 (52)	1386709 (52)

**Educational Attainment#**	Low	60 (27)	62 (28)	68 (28)	62 (25)	47 (22)	59 (27)	358 (26)	(32)
	Intermediate	111 (49)	97 (44)	110 (45)	106 (43)	111 (52)	104 (47)	639 (47)	(43)
	High	55 (24)	61 (28)	65 (27)	76 (31)	56 (26)	57 (26)	370 (27)	(25)

**Elevated cholesterol level**	Yes	48 (21)	43 (20)	37 (15)	42 (17)	39 (18)	38 (17)	247 (18)	Data not available
	No/Don't know	178 (79)	177 (80)	206 (85)	202 (83)	175 (82)	182 (83)	1120 (82)	

**Myocardial Infarction**	Yes	14 (6)	10 (5)	11 (5)	9 (4)	10 (5)	14 (6)	68 (5)	Data not available
	No/Don't know	212 (94)	210 (95)	232 (95)	235 (96)	204 (95)	206 (94)	1299 (95)	

**Household Income (DKK1,000)**	0–200	75 (33)	55 (25)	69 (28)	53 (22)	65 (30)	66 (30)	383 (28)	Data not available
	200–400	52 (23)	55 (25)	69 (28)	55 (23)	52 (24)	61 (28)	344 (25)	
	400–600	37 (16)	47 (21)	53 (22)	63 (26)	32 (15)	41 (19)	273 (20)	
	600-	32 (14)	38 (17)	27 (11)	43 (18)	33 (15)	33 (15)	206 (15)	
	Don't know/denied response	30 (13)	25 (11)	25 (10)	30 (12)	32 (15)	19 (9)	161 (12)	

* [20]

# Data on educational attainment for the Danish population covers age 40–69 years. Data on the population aged 70+ are not available

**Table 2:** Consent to therapy and self reported understanding of information given. Percentages in parentheses

	**Postponement of heart attack**						
	1 month	6 months	12 months	2 years	4 years	8 years	Total
**Consent to therapy**							
Yes	88 (39)	115 (52)	135 (56)	156 (64)	143 (67)	160 (73)	797 (58)
No	112 (50)	77 (35)	72 (30)	62 (25)	47 (22)	38 (17)	408 (30)
Too little information	26 (12)	28 (13)	36 (15)	26 (11)	24 (11)	22 (10)	162 (12)

**Level of understanding**							
Not difficult	177 (78)	170 (77)	192 (79)	210 (86)	174 (81)	179 (81)	1102 (81)
A little difficult	35 (16)	32 (15)	37 (15)	29 (12)	26 (12)	28 (13)	187 (14)
Very difficult	11 (5)	11 (5)	8 (3)	4 (2)	6 (3)	11 (5)	57 (4)
Impossible to understand	3 (1)	7 (3)	6 (2)	1 (0)	8 (4)	2 (0)	27 (2)

**Table 3:** Multivariate logistic regression analysis of the odds for consenting to therapy. (0 = "no" or "uncertain", 1 = "yes", N = 1,367)

**Variable**	**Odds ratio**	**95% Confidence interval**	*p*
**Intervention effectiveness **(Log (base 2) of years postponement of myocardial infarction)	1,266	1.198;1.338	**<0.001**
**Extended information or not **(with or without presentation of baseline risk)			
No (=0)	1	-	
Yes (=1)	0.690	0.548;0.869	**0.002**

**Level of understanding**			
Not difficult	1	-	**-**
A little difficult	0.488	0.349; 0.682	**<0.001**
Very difficult	0.384	0.208; 0.709	**0.002**
Impossible to understand	0.239	0.100;0.571	**0,001**

**Elevated cholesterol level**			
No/don't know (=0)	1	-	
Yes (=1)	1.852	1.751:2.577	**<0.001**

**Myocardial infarction**			
No/don't know (=0)	1	-	**-**
Yes (=1)	2.527	1.276;5.000	**0.008**

**Age**			
40–49	1	-	**-**
50–59	1.554	1.138;2.124	**0.006**
60–69	1.373	0.985;1.912	0.061
70–79	1.836	1.265;2.664	**0.001**
80+	0.965	0.615;1.514	0.876

**Educational attainment**			
Low	1	-	-
Intermediate	1.053	0.789;1.406	0.725
High	0.959	0.693;1.326	0.801

**Gender**			
Male (=0)	1	-	**-**
Female (=1)	0.770	0.610;0,971	**0.027**
